# Health Warnings on Tobacco Packages: A Compliance Assessment Study Around Educational Institutions in Bhubaneswar, India

**DOI:** 10.7759/cureus.51206

**Published:** 2023-12-28

**Authors:** Nancy Satpathy, Pratap K Jena, Venkatarao Epari, Amit Yadav, Shubharanjan Jena, Smruti Priyambada Pradhan, Samarendra Dash

**Affiliations:** 1 Community Medicine, Siksha 'O' Anusandhan Deemed to be University Institute of Medical Sciences and SUM Hospital, Bhubaneswar, IND; 2 Public Health, Indian Council of Medical Research, New Delhi, IND; 3 Health Care Management, Swiss School of Business and Management (SSBM) Geneva, Geneva, CHE; 4 School of Public Health, Kalinga Institute of Industrial Technology (KIIT) Deemed to be University, Bhubaneswar, IND; 5 Tobacco Control, International Union Against Tuberculosis and Lung Disease (The Union) South East Asia Office, New Delhi, IND; 6 Orthodontics and Dentofacial Orthopaedics, National Aluminium Company Limited (NALCO), Bhubaneswar, IND

**Keywords:** tobacco, cotpa, vendors, health warnings, compliance

## Abstract

Background

Graphic health warning labels (HWLs) on tobacco product packaging have been identified by the World Health Organization (WHO) as a cost-effective policy intervention to warn consumers about the health risks of tobacco. Compliance with HWLs shields young individuals from tobacco marketing influences and exposes users to health warnings. Assessing compliance with health warning labels would provide insights into the state of law implementation. The study assessed health warning labels on tobacco packages as per the Cigarettes and Other Tobacco Products (COTPA) (Packaging and Labelling) Amendment Rules of 2020, specifically assessing their availability around educational institutions in Bhubaneswar, Odisha, India.

Materials and methods

From August 2022 to January 2023, a protocol was employed to collect information on the sale of tobacco products around educational institutions including packages of cigarettes, beedis, and smokeless tobacco (SLT) from Bhubaneswar City. Using multistage random sampling 18 schools were selected in Bhubaneswar City. Areas within 100 yards (91.44 meters) of each school were mapped using a map tool. All prospective tobacco vendors within 100 yards of each school were included in the study. The data on compliance with HWLs were summarized using descriptive statistics. The health warnings compliance assessment of the tobacco products available with the vendors was conducted using three major indicators, including analysis of the font content, size and element of the graphics, and textual health warnings. In addition, the surface area occupied by these warnings was measured using a calibrated ruler.

Results

Within 100 yards of 18 schools in Bhubaneswar city, 57 vendors were found selling tobacco. About 48 distinct brands and 791 unbranded tobacco products were identified from 2135 packets collected from 57 vendors. Out of the 48 branded product packets examined, 25 brands were for smoking (cigarettes, bidis), while 23 were for SLT products such as khaini, gutkha, and pan masala containing tobacco. Only six brands out of 17 cigarette packs complied with HWL provisions. None of the eight unique bidi packs and 302 unbranded bidi packs were compliant with any HWL compliance indicators. Other compliance-related issues included incomplete health warning labels, out-of-rotation pictorial health warnings, distorted printing (blurry, heavy tint, and faded), and split warnings.

Conclusion

Tobacco products were sold within 100 yards of educational institutions in clear violation of the COTPA Section 6 provisions. Furthermore, the tobacco products sold were also not in compliance with the health warning label laws under section 7 of COTPA. There is an urgent need for strict enforcement of the provisions relating to the ban on sale within 100 yards of educational institutions and health warning label rules in Bhubaneshwar.

## Introduction

Tobacco kills more than 7 million of its users prematurely and approximately 1.2 million nonusers due to environmental tobacco smoke [1]. India is the world's third-largest producer of tobacco products, and it has the world's fifth-largest international trade and second-largest consumer [2,3]. India has approximately 267 million (28.6%) adult tobacco users [3], which cost more than 1.04 percent of India's Gross Domestic Product (GDP) in 2017-18 [4]. The economic impact of tobacco use among Indian adults in 2017-2018 amounted to INR 1773.4 billion (US $27.5 billion), with 22% being direct costs and 78% as indirect expenses [5].

The World Health Organization (WHO) Framework Convention on Tobacco Control (FCTC) and the national tobacco control law in India, the Cigarettes and Other Tobacco Products Act (COTPA) have mandated several evidence-based measures to address the tobacco epidemic. The WHO introduced six MPOWER (M: monitor tobacco use and prevention policies; P: protect people from tobacco smoke; O: offer help to quit tobacco smoking; W: warn about the dangers of tobacco; E: enforce bans on tobacco advertising, promotion, and sponsorship; and R: raise taxes on tobacco) measures to assist countries in implementing key measures of the WHO Framework Convention on Tobacco Control (FCTC) [6, 7]. The WHO FCTC as well as National Tobacco Control laws require all tobacco products to bear graphic health warning labels (HWLs). They have recognized HWLs on all tobacco products as a cost-effective tool for notifying the vast population about the health dangers associated with consumption/exposure to tobacco [8]. HWLs, as per COTPA (enacted in 2016), are required to cover 85 percent of the main display area on both sides of all tobacco packages, with 25% dedicated to text warning and quit-line numbers and 60% dedicated to the pictorial health warning. There is one photo for the first 12-month rotation period and another photo for the second 12-month rotation period [9-12].

For school students, impactful health warnings play a crucial role in shaping perceptions and decisions regarding tobacco use, discouraging experimentation, initiation, and habit formation [13]. Ensuring visible and compliant health warnings fosters environments that actively discourage tobacco use among youth, influencing healthier choices and attitudes towards tobacco [14].

A few studies have investigated health warnings on tobacco pack compliance with regard to COTPA's prescribed Section 7 and 8 rules for tobacco products sold in India. A study by Saraf et al. (2021) conducted in five Indian states found that only one percent of the smokeless tobacco products and none of the bidi packets were compliant with the location, content, and size of HWL [15]. Another study in the Udupi district of Karnataka, India, found that compliance was zero percent for local companies, 6.7% for national companies, and 93.3% for multinational companies [16]. A scoping review study conducted by Mudey et al. (2023) evaluates the impact of graphic health warnings on tobacco packaging in India and suggests the need for more visible and comprehensible images to effectively discourage tobacco use [17]. A comprehensive community-based cross-sectional analytical research study by Joseph et al. (2021) using 2044 tobacco product packs gathered from various points of sale discovered that a higher proportion of smokeless/local variety tobacco products lacked a health warning label [18].

Given the prevalence and pattern of tobacco use in India, thorough, comprehensive compliance assessment studies are essential for determining the present situation of packaging law implementation. Odisha, an Indian state from the eastern region, has a higher prevalence rate of tobacco use (45.6%) among adults and 6.2% of school students [3, 19]. Nearly half of current cigarette and bidi smokers obtain their products from vendors, with 34% of cigarette smokers and 12% of bidi smokers not being refused due to their age [20]. As per COTPA 2003, Section 6 prohibits the sale of cigarettes and other tobacco products to anyone below the age of 18 years and in an area within a radius of 100 yards (91.44m) of any educational institution. Hence, the study aimed to evaluate the census of all tobacco vendors and the compliance of tobacco packs marketed around 100 yards of educational institutions in the Bhubaneswar smart city of Khordha district in Odisha with the provisions in COTPA specific to health warnings.

## Materials and methods

This study is part of a doctoral research study on compliance with Point-of-Sale provisions under the COTPA and student tobacco use in Bhubaneswar City, India. The study was conducted from August 2022 to January 2023. The city has been geographically divided into three Bhubaneswar Municipal Corporation (BMC) zones: north, southeast, and southwest [21]. Among the high schools listed under the Department of School and Mass Education, Government of Odisha, there are a total of 124 schools spread across three zones. Out of 124 schools, 18 were included in the study (approximately 15%). Six schools were randomly selected from each zone using a random table. The inclusion criteria involved the selection of vendors within a 100-yard (91.44m) radius of each of the 18 chosen schools.

Ethical clearance was obtained from the institutional ethics committee of Siksha ‘O’ Anusandhan, Deemed to be University (Letter No.: Ref. No./DMR/IMS.SH/SOA/2021026), Bhubaneswar, India.

**Figure 1:**
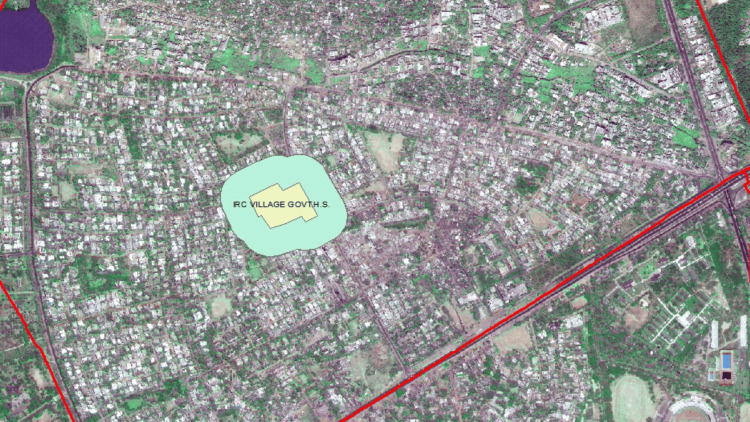
Mapping of the 100 Yard Radius Around School. ArcMap 10.8 map tool was used to locate the 100-yard radius around the schools and the major locations or landmarks in the marked radius. The school in the above picture was not selected in the study.

The vendors within 100 yards of the selected schools were identified using the mapping software ArcMap 10.8. A radius of 100 yards was drawn from the boundary of each school, and all prospective vendors with the potential and scope for tobacco sales were included in the study. Google Maps and satellites were also utilized to find significant landmarks, areas, and roads (Figure 1). Every individual vendor was chosen. The types of vendors sampled were determined by the Global Youth Tobacco Survey (GYTS) [19], the Global Adult Tobacco Survey (GATS) [3], and input from local partners. These included small grocery stores, paan bidi shops, street vendors, and tobacco specialists. For every brand sold, a distinct tobacco pack was obtained. The vendors that were observed to be closed after two repeated visits were excluded from the study.

One empty pack from each variety of tobacco products was obtained from each vendor. In India, the sale of loose cigarettes is widespread, with cigarettes sold from cigarette packs until the pack is empty. So, the shopkeeper used to store the empty packs, stock the empty packs, and discard them one at a time. In the absence of an empty pack, a fresh pack was purchased. One packet of each brand of tobacco product accessible in vendors was purchased to ensure that all tobacco brands were represented accurately and without duplication. Unbranded packs were excluded from the analysis as they violate the Legal Metrology (Packaged Commodities) Rules, 2011 [22], and COTPA 2003.

**Figure 2:**
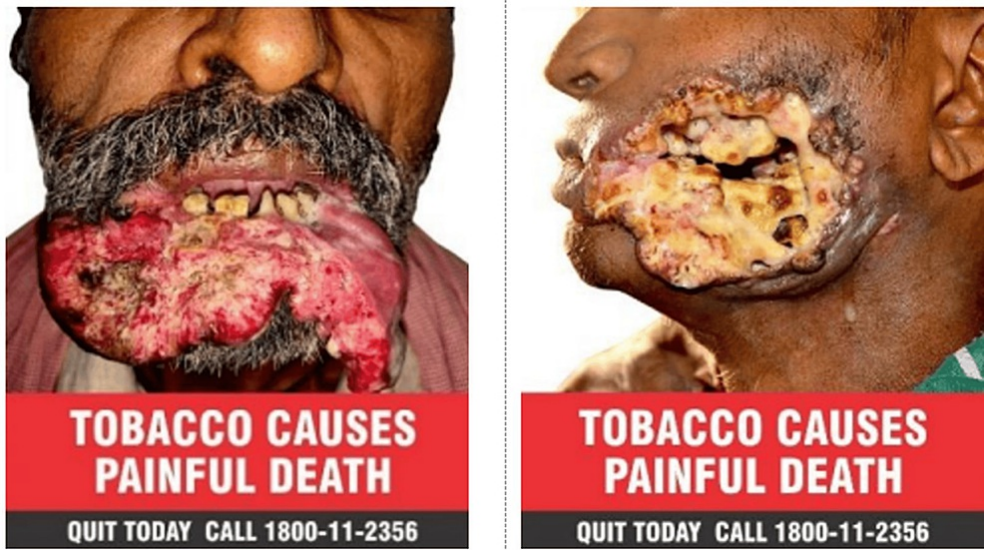
Reference picture as per the amendment specified in notification number G.S.R. 693(E) 2020

Visual analysis of tobacco packets was done by observing font content, size, and elements of the graphics and textual health warnings. I) For font content on: (1) Textual Health Warning: For all forms of tobacco, smoking and smokeless, the words “TOBACCO CAUSES PAINFUL DEATH” shall appear in white font colour on a red background, and the words “QUIT TODAY CALL 1800-11-2356” shall appear in white font colour on a black background; “WARNING” shall be in white font colour on a red background, and product-specific warnings shall be in white font colour on a black background with no textual and not relevant textual warning. (2) Pictorial Health Warning: At present, the required pictorial warning image was first the frontal view of a male face with mouth cancer on the right side and second the frontal view of a male neck with throat cancer. Packages with at least one in-rotation product-specific HWL were evaluated for compliance (as specified for the last 12 months of the 2020-22 cycle); observation for out-of-rotation pictorial warning, no pictorial warning, and no relevant pictorial warning. II) For size: HWL size covers 85% of the visible principal display area covered by the HWL, with 60% covered by the HWL graphic and 25% covered by the HWL text. III) For health warning label elements, the elements must be free of distortion and complete (comprehensible and well-defined HWLs). Additional elements noted for more product specification details include the language used (English/Hindi/Odia/others), cigarette packs for bevel, and bidi and smokeless tobacco (SLT) headshot displays. The evaluation of compliance with health warning requirements is conducted according to the Ministry of Health and Family Welfare Notification General Statutory Rules (G.S.R.) 182(E) [10] issued in 2008, 454(E) [11] issued in 2020, and the amendment specified in notification number G.S.R. 693(E) [12] issued in the same year (Figure 2). A visual analysis of the product specification details and specific health warnings (SHWs) was conducted, and the surface area occupied by these warnings was calculated with the help of a calibrated ruler.

## Results

Around 100 yards from 18 schools in three zones of the Bhubaneswar Municipal Corporation (BMC), 68 vendors were surveyed. After two repeated visits, 17 vendors were excluded from the study as they were observed to be closed. A total of 57 vendors across 18 schools were found selling tobacco, of which 32 were small grocery stores; 18 were paan and bidi shops; five were street mobile vendors; and two were tobacconist shops. Among the 295 cigarette packets, 17 unique brands were identified. Among 453 bidi packets, 8 unique brands and 302 unbranded packets were identified, and among 1387 smokeless (khaini, gutkha, and pan masala with tobacco) packets, 23 unique brands and 489 unbranded packets were identified. The study took samples of 48 different brands of tobacco products from 2135 packets of tobacco.

**Figure 3:**
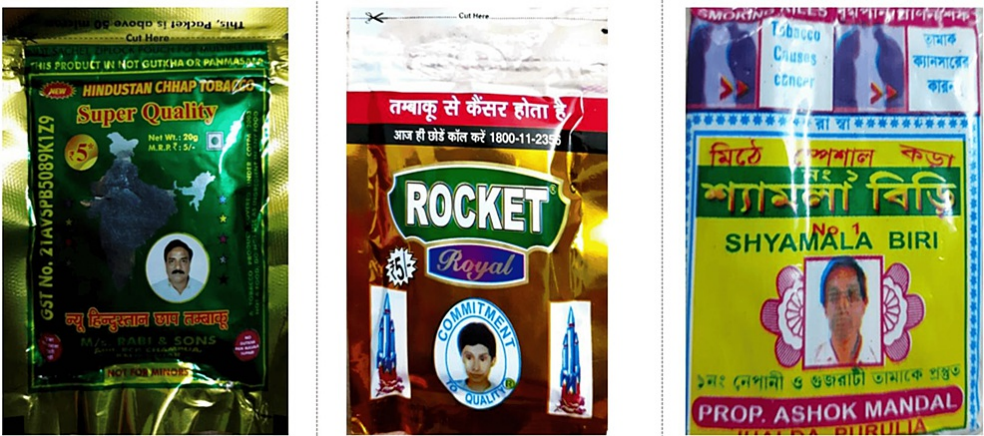
Examples of Non-Complaint warning packs (no/no relevant textual health warnings)

**Figure 4:**
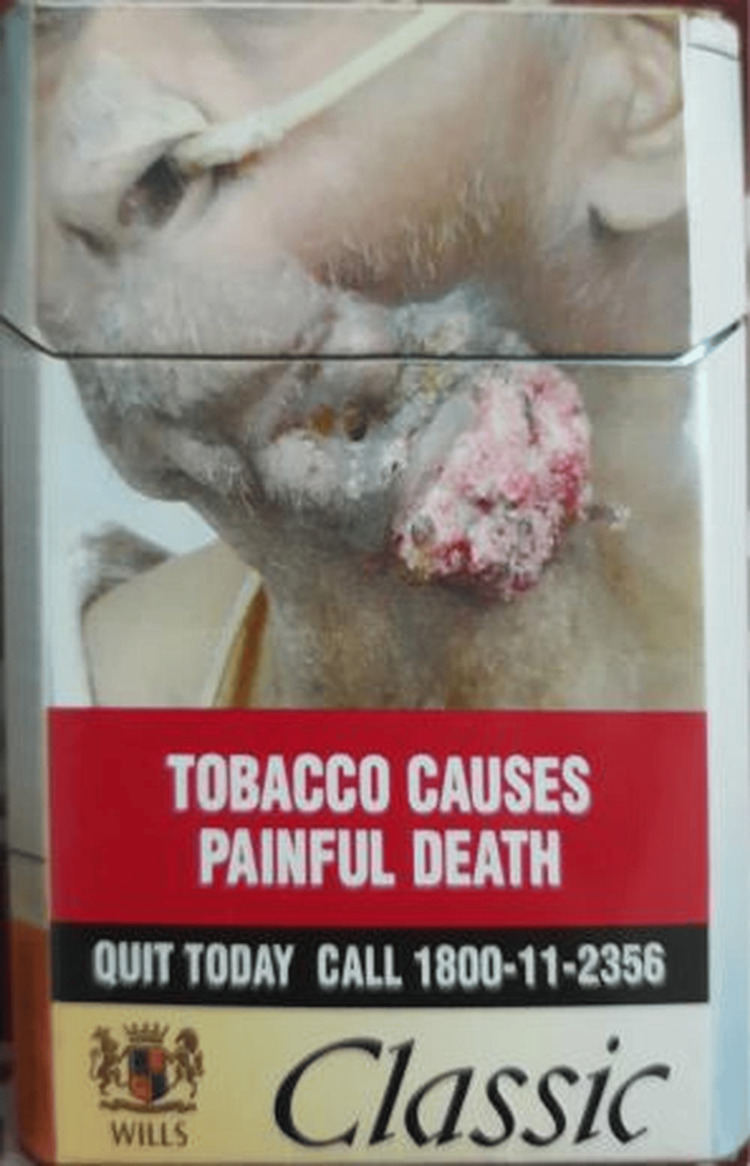
Out-of-rotation pictorial health warning

**Figure 5:**
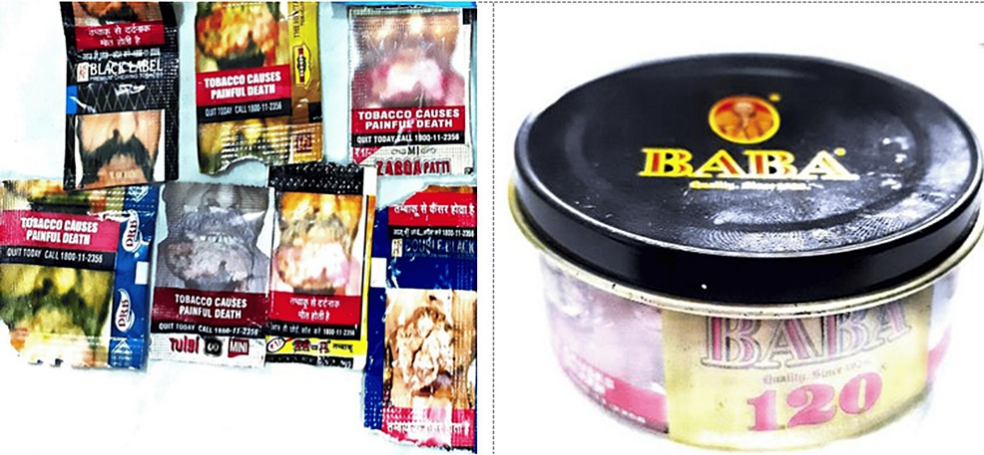
Distorted (blurry, heavy tint and faded) and split warning making the pictorial warning appear unclear

Only 27.1% (13/48) of tobacco-branded packs had textual warnings, whereas none of the bidi packets had any recent textual warnings. There were no relevant textual health warnings on 72.9% (35/48) of all tobacco-branded packs (Figure 3). Nearly 41 packs (85.4%) had out-of-rotation pictorial warnings (Figure 4), no pictorials, or no relevant pictorial health warnings (Figure 3). Approximately 37.5% of all branded tobacco packs contained no pictorial health warnings. Most SLT and bidi packs had blurred/faded/heavy tint/distorted/split images as pictorial warnings (Figure 5).

**Figure 6:**
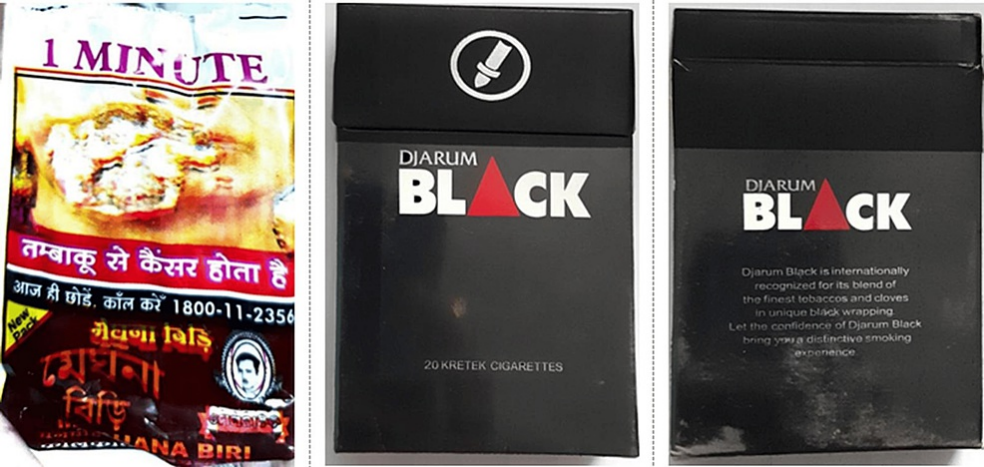
No Legible and Prominent/No Textual and Pictorial warning written on both panels

**Figure 7:**
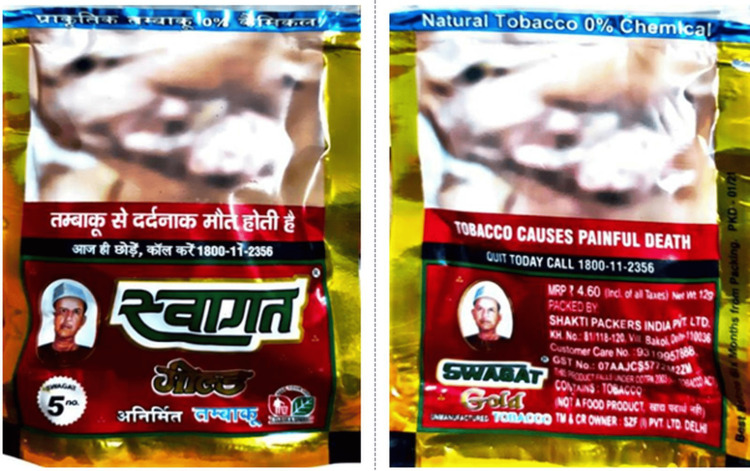
Textual and pictorial health warning coverage of principal display area - less than 85%

**Figure 8:**
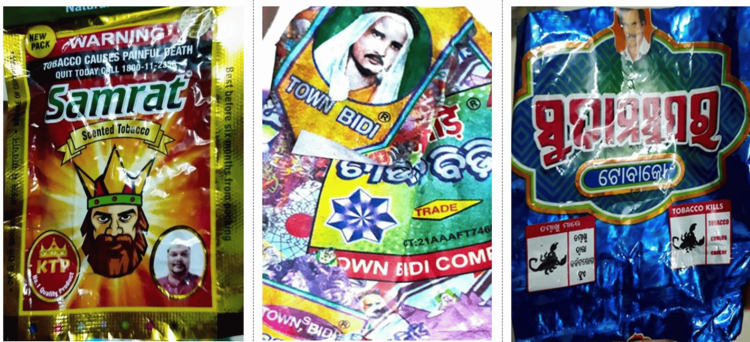
Headshot for Smokeless Tobacco and Bidi

Only 14.6% of the packs had visible and legible textual and pictorial warnings, while the remaining packs either had no textual/pictorial warnings or both (Figure 6). The total area of the Specified Health Warning (SHW) met the prescribed size of 85% of the principal display area on only seven brands (six cigarette brands and one SLT brand) (Figure 7). Bevel edges were found on more than 30% of cigarette packs. Eleven (23%) bidi and SLT packs had included headshots of men and children, many of whom were dressed in western-style business wear (jacket and tie), though there were also some photos of men in more traditional dress and a few had children's pictures in them (Figure 8).

Among the 17 cigarette packs collected, only six brands completely comply with HWL provisions. All eight unique bidi packets didn’t comply with the HWL provisions under COTPA. Out of 23 brands, only one brand complies with the HWL specifications. And finally, out of 48 unique brands, only seven brands comply with all the HWL specifications (Table 1).

**Table 1:** Descriptive Characteristics of the Tobacco Packages

Items	Health Warning Provisions	Cigarette n=17 Brands, n (%)	Bidi n=8 Brands, n (%)	Smokeless Tobacco n=23 Brands, n (%)	Total n=48 Unique brands, n (%)
Textual Health Warning	Have *“*TOBACCO CAUSES PAINFUL DEATH*”* written in white font on a red background and Have *“*QUIT TODAY CALL 1800-11-2356*”* written in white font colour on a black background	9(52.9)	0	4(17.4)	13(27.1)
	No textual warning	2(11.8)	3(37.5)	8(34.8)	13(27.1)
	Not Relevant Textual warning	6(35.3)	5(62.5)	11(47.8)	22(45.8)
Pictorial Health Warning	Have frontal view of a male face with mouth cancer on the right side/frontal view of a male neck with throat cancer	6(35.3)	0	1(4.3)	7(14.6)
	Out-of-rotation pictorial warning	3(17.6)	0	7(30.4)	10(20.8)
	No pictorial warning	2(11.8)	5(62.5)	11(47.8)	18(37.5)
	Not Relevant pictorial warning	6(35.3)	3(37.5)	4(17.4)	13(27.1)
Textual and pictorial health warning coverage of the principal display area	Less than 85%	3(17.6)	0	3(13)	6(12.5)
	85%	6(35.3)	0	1(4.3)	7(14.6)
	Not applicable	8(47.1)	8(100)	19(82.6)	35(72.9)
Language Health Warning Labels	English	17(100)	2(25)	5(21.7)	24(50)
	Hindi	0	3(37.5)	12(52.2)	15(31.3)
	Odia	0	1(12.5)	3(13)	4(8.3)
	Others	0	2(25)	3(13)	5(10.4)
Bevelling	Have bevelling for cigarettes	6(35.3)	Not Applicable	Not Applicable	-
Headshot	Have headshot for Bidi and Smokeless Tobacco	Not Applicable	4(50)	7(30.4)	-

## Discussion

The study's findings reveal that HWL compliance on tobacco packs is comparatively low, with only 27.1% (cigarette and SLT packs) and none of the bidi packs fully complying with all indicators. Very low compliance with proportionate size, legibility, and language criteria for health warnings on tobacco products marketed in Bhubaneswar, Odisha, India. These extremely low compliance rates highlight a need for increased enforcement efforts and execution, given that India has one of the stronger packaging and labeling rules in Southeast Asia [6]. Positioning and large-size health warnings on packaging may increase visibility and effectiveness while reducing space for brand promotional messages. Health warnings on cigarette packaging are a low-cost way to raise public knowledge of the dangers of tobacco use, encourage cessation, and reduce tobacco consumption [23]. To maximize the impact of these HWLs, the government must ensure that manufacturers print warnings on packs in accordance with the law. Similar to this study, another study by Panigrahi and Sharma (2019), which took place in rural Bhubaneswar, Odisha, found that none of the bidi packages included any compliant health warning with appropriate location and coverage [23].

In accordance with a previous study in India, the study identified a high level of non-compliance with warnings on tobacco packs, especially SLT and bidis, which is consistent with previous findings [15, 16, 24, 25]. Tobacco packs have varying sizes and shapes. The GATS (2016-17) found that, compared to cigarettes, fewer users saw HWLs on packets for SLT and bidi. Some SLT packs and bidi packs in our sample were too small to even carry an HWL that fulfilled the minimal specifications. A study by Saraf et al. [15] found that most study samples were hand-wrapped in conical paper packets with curved surfaces and that curvatures distort the image, preventing full exposure to the warning message. The lack of a standard shape and size limits the applicability of HWLs. A consistent shape and size could strengthen the warnings' significance and impact. Additionally, it is critical to examine the issues associated with insufficient or deceptive HWLs. Regarding the split HWLs on the SLT packs, it is possible that this is a result of defective imprinting by the local manufacturers. It is vital to monitor these manufacturers' performance on a regular basis to guarantee that the image printing quality is maintained as per COTPA prescriptions.

The study discovered a 21% presence of headshots on Bidis and SLT products, which is against the law. Smith et al. [26] shed some light on how headshots contribute to the branding strategy for bidi and SLT. They may be factual (the photo may reflect the owner of the company that created the product), aspirational (the photo may depict the type of person who would use it), or both. The faces could be used as an informal trademark. Nonetheless, they propose that portrayals of potentially aspirational or emotive entities, particularly deities, children, and babies, should be restricted in future marketing and packaging limitations.

Furthermore, the study revealed a significant lack of compliance with Section 6b of COTPA, which prohibits the sale of tobacco products within a 100-yard radius of educational premises [24]. All 18 selected schools in the study witnessed the presence of tobacco products in their vicinity. Similarly, numerous studies conducted in various Indian states have also documented violations of Section 6b [27-30].

The study has a few limitations. The study was conducted in one zone of one Indian state, and compliance with COTPA, or enforcement measures, may vary by region. Also, just 48 tobacco brands were evaluated for health warning compliance, which may not be reflective of all tobacco brands. To achieve proper health warning compliance, manufacturers, distributors, and retailers of tobacco products must strictly follow COTPA requirements, and the restrictions must be enforced.

## Conclusions

To communicate the detrimental effects of tobacco use to consumers and non-consumers, health warnings are one of the most cost-effective approaches. According to study findings, compliance with the National Legislation-COTPA is extremely low in the area studied, necessitating the adoption of strong actions to ensure that the health warnings on tobacco product packages are in compliance with the law. Furthermore, the study revealed inadequate implementation of Section 6b of COTPA in Bhubaneswar. Addressing this issue necessitates a concerted effort from various stakeholders, including law enforcement agencies, educational institutions, the community, and relevant authorities. It is crucial to enhance public awareness regarding the detrimental consequences of tobacco use by promoting voluntary compliance with regulations. Embracing the guidelines for Tobacco-Free Educational Institutions (TOFEI) issued by the Ministry of Health and Family Welfare, can foster a comprehensive approach to tobacco control and enhance the implementation of COTPA within and in the vicinity of educational establishments not only in Bhubaneshwar but across the country in India.
